# Genotyping *Cryptosporidium andersoni* in Cattle in Shaanxi Province, Northwestern China

**DOI:** 10.1371/journal.pone.0060112

**Published:** 2013-04-01

**Authors:** Guang-Hui Zhao, Wan-Xin Ren, Man Gao, Qing-Qing Bian, Bing Hu, Mei-Mei Cong, Qing Lin, Rong-Jun Wang, Meng Qi, Mao-Zhen Qi, Xing-Quan Zhu, Long-Xian Zhang

**Affiliations:** 1 State Key Laboratory of Veterinary Etiological Biology, Key Laboratory of Veterinary Parasitology of Gansu Province, Lanzhou Veterinary Research Institute, Chinese Academy of Agricultural Sciences, Lanzhou, Gansu Province, China; 2 College of Veterinary Medicine, Northwest A&F University, Yangling, Shaanxi Province, China; 3 Pulike Biological Engineering Inc., Luoyang, Henan Province, China; 4 College of Animal Science and Veterinary Medicine, Henan Agricultural University, Zhengzhou, Henan Province, China; Thomas Jefferson University, United States of America

## Abstract

The present study examined the prevalence and genotypes of *Cryptosporidium andersoni* in cattle in Shaanxi province, China. A total of 2071 fecal samples (847 from Qinchuan cattle and 1224 from dairy cattle) were examined for the presence of *Cryptosporidium* oocysts, and 70 samples (3.4%) were *C. andersoni*-positive and those positive samples were identified by PCR amplification of the small subunit ribosomal RNA (SSU rRNA) and the *Cryptosporidium* oocyst wall protein (COWP) genes. *C. andersoni* was the only species found in the examined cattle in this province. Fifty-seven *C. andersoni* isolates were characterized into 5 MLST subtypes using multilocus sequence typing analysis, including a new subtype in the native beef breed Qinchuan cattle. All of these *C. andersoni* isolates presented a clonal genetic structure. These findings provide new insights into the genetic structure of *C. andersoni* isolates in Shaanxi province and basic data of *Cryptosporidium* prevalence status, which in turn have implications for controlling cryptosporidiosis in this province.

## Introduction

*Cryptosporidium* spp. infect a wide range of hosts including humans and cattle [1], [2]. Previous studies around the world have shown that cattle is the most common species of mammals known to be infected with *Cryptosporidium* spp., and is the main source of human infection with *Cryptosporidium* [2]–[4]. Four *Cryptosporidium* species, namely *C. parvum*, *C. andersoni*, *C. bovis* and *C. ryanae,* can infect cattle and cause bovine cryptosporidiosis [5]. Among them, *C. andersoni* has been identified to be the predominant species responsible for cattle infection in China [6]. There are subclinical signs in cattle infected with *C. andersoni* associating with poor weight gain and reduce of milk yield [7], [8]. *C. andesoni* also has been isolated from humans with diarrhoea in England [9] and paediatric patients in Malawi [10].

Traditionally, identification of *Cryptosporidium* spp. is based on morphologic examination, mainly using Sheather's sugar flotation technique [11] and modified acid-fast staining method [12], but this approach is not reliable for delineating *Cryptosporidium* species because of their morphologic similarities [13]. Alternatively, molecular tools are useful for the accurate identification of *Cryptosporidium* species and better understanding of population genetics of *Cryptosporidium*, which have important implications for studying their pathogenesis and clinical presentations [14], [15]. The small subunit ribosomal RNA (SSU rRNA) gene [16] and the *Cryptosporidium* oocyst wall protein (COWP) gene [17]–[19] have been used successfully as genetic markers for the identification of *Cryptosporidium* species and genotypes in hosts and environmental (water and food) samples. But genotyping tools have limitations in some epidemiologic investigations as a result of the low resolution power of these loci [17]. Consequently, several subtyping tools have been developed for molecular epidemiologic studies of *Cryptosporidium* spp. Multilocus sequence typing (MLST), based on both length polymorphism and single nucleotide polymorphism (SNP), is a high-resolution typing tool that can accurately describe genetic diversity of parasites [20]. Recently, *C. muris* and *C. andersoni* subtypes were successfully analyzed by MLST targeting microsatellite and minisatellite sequences [21], [22].

In China, *Cryptosporidium* infection has been reported in dairy cattle in Guangxi [23], Anhui [24], Henan [6], [25] and Qinghai provinces [26], and in beef cattle in Anhui [27], Qinghai [26], Inner Mongolia [28] and other provinces. However, these studies mainly focused on prevalence and identification of *Cryptosporidium* species. Qinchuan cattle, originated in Guanzhong Plain in Shaanxi province, is a famous native beef cattle breed in China, which now has been introduced to more than 20 provinces in China. However, there is no publication about *Cryptosporidium* infection in Qinchuan cattle except prevalence of other intestinal parasites reported by our group [29]. Here, the prevalence of *C. andersoni* infection in Qinchuan cattle in Shaanxi province was investigated and compared with that in dairy cattle in this province. The subtypes of *C. andersoni* in Qinchuan cattle and dairy cattle in this province were also characterized using MLST.

## Materials and Methods

### Ethics Statement

The performance of this study was strictly according to the recommendations of the Guide for the Care and Use of Laboratory Animals of the Ministry of Health, China, and our protocol was reviewed and approved by the Research Ethics Committee of Northwest A&F University. All the fecal samples were collected from animals after the permission of farm owners, with no specific permits being required by the authority for the collection of fecal samples.

### Specimen Collection and Examination

Between November 2010 and May 2012, a total of 2071 fecal samples from intensively reared pre-weaned calves (3 weeks −<3 months old), post-weaned calves (3–11 months old), heifers (1–2 years old), and adult cattle (>2 years old) were obtained directly from the rectum of each animal using sterile disposable gloves, then placed in clean plastic bags labeled with the animal’s breed, age, and geographical origin (Table 1) in Shaanxi province, China. These samples were then examined by Sheather's sugar flotation technique and microscopy at 400×magnification [11]. *Cryptosporidium*-like samples were kept in 2.5% potassium dichromate solution before DNA extraction.

**Table 1:** Factors associated with prevalence of *Cryptosporidium andersoni* genotypes in cattle in Shaanxi province, Northwestern China.

Factors		Qinchuan cattle						Dairy cattle			
		Sample size	No. positive (%)	No. subtypes*				Sample size	No. positive (%)	No. subtypes*	
				**A**	**B**	**C**	**D**			**A**	**E**
Collection site	Yangling district	561	33 (5.88)	20	3	1	1	445	11 (2.47)	1	6
	Tongchuan city	84	0	0	0	0	0	188	8 (4.26)	0	8
	Mei county	138	0	0	0	0	0	78	2 (2.56)	0	2
	Qian county	34	5 (14.71)	5	0	0	0	143	6 (4.20)	0	6
	Dali county	0	0	0	0	0	0	86	0	0	0
	Shenmu county	0	0	0	0	0	0	14	0	0	0
	Xi 'an city	30	0	0	0	0	0	270	5 (1.85)	0	4
	Total	847	38 (4.49)	25	3	1	1	1224	32 (2.61)	1	26
Age group	<3 month	14	3 (21.43)	3	0	0	0	119	0	0	0
	3–11 months	67	17 (25.37)	11	1	1	0	250	11 (4.40)	0	10
	1–2 year	447	14 (3.13)	9	2	0	1	224	8 (3.57)	0	7
	>2 year	319	4 (1.25)	2	0	0	0	631	13 (2.06)	1	9
	Total	847	38 (4.49)	25	3	1	1	1224	32 (2.61)	1	26

Note: *A, B, C, D, E respresent subtypes A4, A4, A4, A1; A2, A4, A4, A1; A2, A4,A2, A1; A4, A4, A2, A1; A1, A4, A4, A1, respectively.

### DNA Isolation and PCR Amplification

The positive samples were washed extensively in distilled water to remove the potassium dichromate solution. DNA was extracted from each microscopically positive sample using the E.Z.N.A.® Stool DNA Kit (OMEGA) according to the manufacturer’s instructions, and stored at −20°C until further processed. *Cryptosporidium* species and genotypes were determined by nested PCR amplification of the small subunit ribosomal RNA (SSU rRNA) gene [16] and by single PCR amplification of the *Cryptosporidium* oocyst wall protein (COWP) gene [30]. Subtyping was achieved by amplifying the minisatellite targets (four loci, namely CM-MS_1_ coding for hypothetical protein; CM-MS_2_ coding for 90 kDa heat shock protein; CM-MS_3_ coding for hypothetical protein; CM-MS_16_ coding for leucine rich repeat family protein) according to Feng et al [21] and Wang et al [22]. DNA of *Cryptosporidium andersoni* was used as the positive control for each target gene-based PCR analyses, and samples without DNA and host (cattle) DNA were included in each amplification run to exclude contamination. The primers used in PCR analysis of all gene targets, annealing temperatures, and sizes of the expected PCR products are listed in Table 2. Amplification products were examined by 1.5% agarose gel electrophoresis and stained with ethidium bromide.

**Table 2:** Primers used in the study, annealing temperatures used in the PCR and expected sizes of the PCR products.

Gene	Primer	Sequence (5'–3')	Annealing temperature (°C)	Fragment length (bp)	References
SSU rRNA	F1	CCCATTTCCTTCGAAACAGGA	55	830	[14]
	R1	TTCTAGAGCTAATACATGCG			
	F2	AAGGAGTAAGGAACAACCTCCA	58		
	R2	GGAAGGGTTGTATTATTAGATAAAG			
COWP	F	TGTAGCGTTTTCTCCACCTGATAAA	56	450	[27]
	R	GTTGTGTTGATGCGGTGTTC			
CM-MS1	F1	ACCATCTAGAGATAACGAGCGA	55	550	[19]
	R1	GAATCAGAAGATGAGCGACAA			
	F2	CGTGATAGTGGGTATGAATTGGACA	55		
	R2	CGACTGCGATACTCACGTCCT			
CM-MS2	F1	TTGCAACTGTACCTAAATTAGTA	55	457	[19]
	R1	GTGAGACTTCTGGGGTCCTGA			
	F2	TCATGACGCGTCATACCAACA	52		
	R2	ACTTAGACAGTTCTATGCTGA			
CM-MS3	F1	AACCAAGTGAATCACGAACTT	55	536	[19]
	R1	TCAAGTACAGCAGTCTATTGCTT			
	F2	GCAATATCTTCGACGATCCCA	55		
	R2	ATGGGAATAATTCTTCATCATCAA			
CM-MS16	F1	GAAGAGGTCGAAGTTAAGCTA	50	597	[19]
	R1	GACAATCATCTAAATCGTGTT			
	F2	AAGTTTCATCTAGGTACACTAAGA	55		
	R2	CACTACCTAATCTCGTGTACTT			

### Statistical Analysis and Sequence Analysis

The prevalence of *Cryptosporidium* oocysts and the factors of breed, collection origins and ages were evaluated using Regression Analysis in Statistic Package for Social Science (SPSS) for Windows with 95% confidence intervals (CI). Probability levels (*P*) of <0.05 were regarded as statistically significant.

The positive PCR products were sent to Shanghai Sangon Biological Engineering Biotechnology Company for sequencing using ABI 377 automated DNA sequencer (BigDye Terminator Chemistry) to identify the species/genotype and subtype. The *Cryptosporidium* nucleotide sequences obtained were aligned with reference sequences from the GenBank™ database using the BLAST (http://www.ncbi.nlm.nih.gov) and computer program Clustal X 1.83 [31]. Phylogenetic analysis based on SSU rRNA and COWP gene sequences were conducted to identify species of *Cryptosporidium* isolates in the present study. Neighbor-joining (NJ) method was carried out using Phylip 3.64 [32] with the Kimura two-parameter model. The consensus tree was obtained after bootstrap analysis, with 1000 replications. NJ analysis based on minisatellite sequences was also used to study the relationships of *C. andersoni* isolates with other *Cryptosporidium* by retrieving the reported sequences available in the GenBank™ by Feng et al. [21] and Wang et al. [22]. The *C. andersoni* subtypes were named according to the repeat characteristics of minisatellite repeats in four genetic loci by Feng et al. [21] (Table 3). The genetic diversity of *C. andersoni* was analyzed using DnaSP version 5.10.01 (http://www.ub.edu/dansp/). Linkage disequilibrium was tested using LIAN version 3.5 (http://adenine.biz.fh-weihenstephan.de/cgi-bin/lian/lian.cgi.pl) by a parametric method for four microsatellite/minisatellite loci. STRUCTURE version 2.3.3 was used to describe the population substructure of *C. andersoni* in allelic variation by *K*-means partitional clustering and the admixture model.

**Table 3:** The repeat characteristics of minisatellite repeats at four genetic loci.

Locus	Targeted repeat
CM-MS1	(TAAAGGGCGAGA)_3_ and (GAACGAGATAGG)_12–17_
CM-MS2	(CCATACCTC)_10–11_
CM-MS3	(TGTTGGTGTTGCTGT)_2_ and (TGCTGCAGCTGC)_2–3_
CM-MS16	(CTTCTTCAT)_12,14_

### Nucleotide Sequence Accession Number

Sequences generated in the present study have been deposited in the GenBank database under accession numbers KC580754-KC580823 (for 18S rRNA), KC580824-KC580893 (for COWP), KC580894-KC580958 (for MS1), KC580959-KC581022 (for MS2), KC581023-KC581090 (for MS3) and KC581091-KC581158 (for MS16).

## Results and Discussion

A total of 2071 fecal samples were collected from Qinchuan cattle (847) and dairy cattle (1224) between November 2010 and May 2012, and were examined for the presence of *Cryptosporidium* oocysts. The results are summarized in Table 1. Of these samples, the prevalence of *Cryptosporidium* infection in Qinchuan cattle in Shaanxi province was 4.49% (38/847), which was lower than that in Anhui province [27], Qinghai province [26] in China and in Korea [33]. The prevalence of *Cryptosporidium* in dairy cattle in Shaanxi province (32/1224, 2.61%) was lower than that in Guangxi [23], Anhui [24], Henan [6], [25], Qinghai [26] in China and in rural areas in Korea [33] and in Mongolia [34]. These differences may be related to the different management systems, the timing of specimen collection and the ecological conditions. The results of the SPSS analysis showed that the prevalence of *Cryptosporidium* in Qinchuan cattle was higher than that in dairy cattle in Shaanxi province, but with no significant differences (*P*>0.05). This indicates that the breed of cattle is not associated with the prevalence of *Cryptosporidium* infection. For Qinchuan cattle, the unique beef cattle in China, the highest *Cryptosporidium* prevalence (14.71%) was found in Qian county, but that of dairy cattle was observed in Tongchuan city, indicating no obvious association with the locations (*P*>0.05). These differences may be attributed to the number of samples examined and the timing of sample collection. An age-related difference in *Cryptosporidium* prevalence was observed in this study (Table 1, *P*<0.05). Compared with other age groups, the highest prevalence was noticed in cattle of 3–11 month old (25.37% for Qinchuan cattle, 4.40% for dairy cattle). This was different from results of previous studies that the overall prevalence of *Cryptosporidium* infection had negative relation with age [17], [35], [36].

All microscopically positive samples were confirmed to be infected with *C. andersoni* by characterizations of the SSU rRNA and COWP genes loci. A BLAST similarity search against NCBI nucleotide sequence database indicated that all the obtained sequences of the SSU rRNA and COWP genes have high identity values (>99%) with *C. andersoni* (GenBank accession numbers HQ009808 and AB514044 for the SSU rRNA and COWP gene fragments, respectively). Phylogenetic analysis using NJ based on the SSU rRNA and COWP gene sequences, respectively, showed that all *Cryptosporidium* positive samples were clustered in the clade *C. andersoni* (Data not shown). These results demonstrated that all cattle-derived *Cryptosporidium* isolates in the present study represented *C. andersoni*. This finding was different from that of previous studies in other geographical origins in China [24], [27], [37], India [37], United States [38]–[40] and Denmark [41], where they showed that *C. parvum* was the predominant species in pre-weaned calves, whereas *C. bovis* and *C. ryanae* usually infected post-weaned calves (frequently in yearlings and adult cattle). However, the results of our study are similar to that of Wang et al. [6] who concluded that *C. andersoni* was the predominant species in post-weaned and adult dairy cattle in China. In the present study, all *Cryptosporidium* isolates were identified as *C. andersoni*, which may be related with cattle ages in that sampled cattle were usually older than 3 months. Santín et al. [39] described that cryptosporidiosis in calves infects usually in the first 2 weeks of life. Therefore, the species and prevalence of *Cryptosporidium* infection in cattle before 2-week-old in this region should be investigated in further studies.

**Figure 1:**
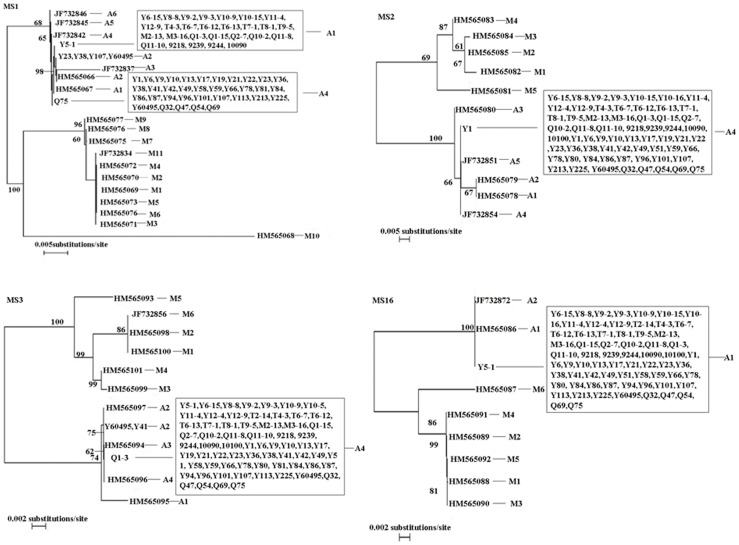
Phylogenetic relationship of *C. andersoni* subtypes based on MS1, MS2, MS3 and MS16 sequences. The trees were reconstructed using respective site sequences of *C. muris* as outgroup and assessed by a neighbor-joining (NJ) analysis of the nucleotide sequences with distance calculated by the Kimura two-parameter model. CM-MS1, CM-MS2, CM-MS3, and CM-MS4 are reference sequences from the whole-genome sequencing project.

Minor intragenotypic variations sometimes can be identified by subtyping tools. At all four minisatellite loci, namely MS1, MS2, MS3 and MS16, a total of 65, 64, 68 and 68 DNA preparations were successfully amplified from each locus, respectively. For *Cryptosporidium andersoni* isolates, 3, 1, 2, and 1 haplotypes were identified at the MS1, MS2, MS3 and MS16 loci, respectively (Figure 1). Two prevalent subtypes were found in the present study: one named subtype A4, A4, A4, A1 herein being derived from 1 dairy cattle and 25 Qinchuan cattle, and the other subtype A1, A4, A4, A1 being unique to dairy cattle. The MLST subtype A2, A4, A4, A1, subtype A2, A4, A2, A1 and subtype A4, A4, A2, A1 were only found in Qinchuan cattle, with 3, 1, 1 isolates for each subtype, respectively (Table 1). Compared with results of Feng et al [21] and Wang et al [22], a new *C. andersoni* MLST subtype (A4, A4, A2, A1) in Qinchuan cattle was identified. In addition, the predominate subtype was subtype A1, A4, A4, A1 for dairy cattle in Shaanxi province, which was different from subtype A4, A4, A4, A1 in other areas of China [22]. These differences may be related to the number of examined specimens and geographic separation.

To determine the presence of the clonal or epidemic genetic structure for *C. andersoni* from cattle in Shaanxi province, the linkage disequilibrium (LD) analysis were performed according to Wang et al. [22] (Table 4). Samples amplified successfully at every locus were included in LD analysis. The standardized index of association (*I^ S^_A_*) was above zero and the pairwise variance (*V_D_*) was greater than the 95% confidence limitation (*L*), indicating the presence of LD and the clonal genetic structure of *C. andersoni* in this province. These results were consistent with *C. andersoni* isolates from other geographical origins in China [22].

**Table 4:** Analysis of linkage disequilibrium in *C. andersoni* from cattle.

Area	No. completely typed	*I^ S^_A_*	*V_D_*	*L*	*P* value
China	99	0.1737	0.7261	0.5256	4.23×10^−33^
Shaanxi province	57	0.0314	0.3431	0.3251	2.55×10^−09^

*I^S^_A_* =  standardized index of association, *V_D_* =  the pairwise variance, *L* = 95% critical value.

**Figure 2:**
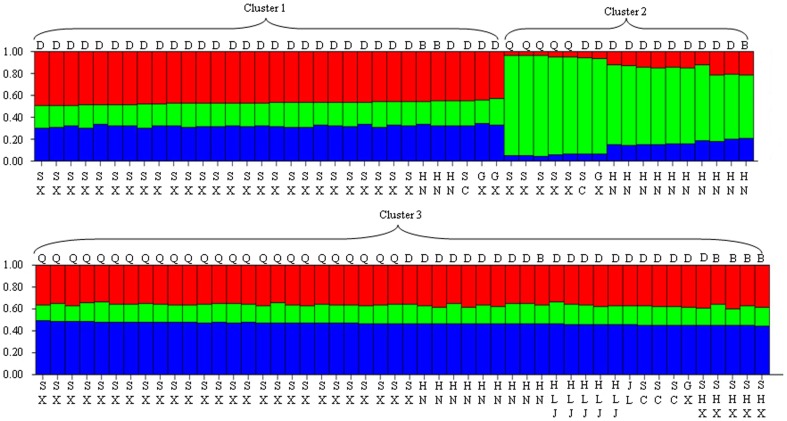
Population structure inferred by Bayesian clustering using multiocus information. A thin vertical line represents each individual, and the geographic regions lie in the bottom. HLJ = Heilongjiang province; SHX = Shanxi province; SX = Shaanxi province; HE = Henan province; JL = Jilin province; SC = Sichuan province; GX = Guangxi province; B = beef cattle; D = dairy cattle; Q = Qinchuan cattle.

The *C. andersoni* samples in China including isolates from Shaanxi province and sequences available in GenBank™ were used in cluster analysis. All the samples formed three clusters (Figure 2). Samples in most provinces except Heilongjiang, Shanxi and Jilin provinces were dispersed in different clusters. All the samples in Shaanxi province were positioned in three clusters, with Qinchuan cattle in cluster 2 and 3, and dairy cattle in cluster 1 and 3. These results were slightly different from finding of Wang et al. [22] in that only two clusters were identified in China, possibly due to the number of sampled specimens and geographical origins. The MLST subtype A1, A4, A4, A1 (n = 32) and A4, A4, A4, A1 (n = 50), the two most common subtypes in cattle in China, located into cluster 1 and cluster 3, respectively. The new MLST subtype A4, A4, A2, A1 was identified within Cluster 2.

Subtyping tools have proven useful for understanding the biologic characteristics of *Cryptosporidium* spp., identifying virulence and clinical presentations among different subtypes, and epidemiological investigation of *Cryptosporidium* species [2], [21], [42]. The 60 kDa glycoprotein (GP 60) gene was the initial subtyping target widely used for studying *C. parvum* and *C. hominis* [41], [42]. However, single locus may be deviated and some genotypes may be missed because of selection of locus and its length. The multilocus typing (MLT) and MLST have been used to genotype *C. parvum* and *C. hominis* [20], [43], [44]. The MLT method may identify the subtype of multiple loci, but sometimes can miss the single nucleotide polymorphisms (SNP), unable to detect some subtypes. Compared with MLT, the MLST tool, which is dependent principally on the genetic heterogeneity by DNA sequencing of the amplified PCR products, can directly and accurately analyze the genotypes and subtypes of *Cryptosporidium* spp. [20]. *C. andersoni* was the preponderant *Cryptosporidium* species in cattle in China [6], resulting in the pathological lesions of the gastric glands and the gastric mucosa, poor weight gain and falling of milk yield [7], [8], [45], [46]. In 2011, a MLST technique for subtyping *C. andersoni* was developed by Feng et al. [21], and used later by Wang et al. [22]. This method can identify successfully the subtypes of *C. andersoni* using the microsatellite and minisatellite markers.

In conclusion, using MLST, the present study identified 5 MLST subtypes among 57 *C. andersoni*-positive specimens from dairy cattle and Qinchuan cattle in Shaanxi province, including a new subtype A4, A4, A2, A1 in the native beef breed Qinchuan cattle. The subtypes A1, A4, A4, A1 and A4, A4, A4, A1 were the prevalent subtypes in dairy cattle and Qinchuan cattle, respectively. *C. andersoni* in cattle in Shaanxi province presented a clonal genetic structure. These findings showed new insights into the genetic structure of *C. andersoni* isolates in Shaanxi province, Northwestern China. The accurate genotyping of *C. andersoni* isolates provided valuable basic data for developing strategies to control *C. andersoni* infection in cattle and evaluate risk of *Cryptosporidium* infection to humans. These findings should have implications for controlling cattle cryptosporidiosis in this province as well as in China.
